# Small RNA Profiling of Influenza A Virus-Infected Cells Identifies miR-449b as a Regulator of Histone Deacetylase 1 and Interferon Beta

**DOI:** 10.1371/journal.pone.0076560

**Published:** 2013-09-26

**Authors:** William A. Buggele, Katherine E. Krause, Curt M. Horvath

**Affiliations:** Department of Molecular Biosciences, Northwestern University, Evanston, Illinois, United States of America; Johns Hopkins University - Bloomberg School of Public Health, United States of America

## Abstract

The mammalian antiviral response relies on the alteration of cellular gene expression, to induce the production of antiviral effectors and regulate their activities. Recent research has indicated that virus infections can induce the accumulation of cellular microRNA (miRNA) species that influence the stability of host mRNAs and their protein products. To determine the potential for miRNA regulation of cellular responses to influenza A virus infection, small RNA profiling was carried out using next generation sequencing. Comparison of miRNA expression profiles in uninfected human A549 cells to cells infected with influenza A virus strains A/Udorn/72 and A/WSN/33, revealed virus-induced changes in miRNA abundance. Gene expression analysis identified mRNA targets for a cohort of highly inducible miRNAs linked to diverse cellular functions. Experiments demonstrate that the histone deacetylase, HDAC1, can be regulated by influenza-inducible miR-449b, resulting in altered mRNA and protein levels. Expression of miR-449b enhances virus and poly(I:C) activation of the IFNβ promoter, a process known to be negatively regulated by HDAC1. These findings demonstrate miRNA induction by influenza A virus infection and elucidate an example of miRNA control of antiviral gene expression in human cells, defining a role for miR-449b in regulation of HDAC1 and antiviral cytokine signaling.

## Introduction

Virus infection of mammalian cells induces immediate and robust changes in cellular gene expression. Detection of virus infection by cellular signaling machinery triggers the transcription of antiviral genes including primary antiviral cytokines in the type I interferon (IFN) family as well as diverse effectors of the antiviral state [1]. These cytokines and antiviral genes also drive further gene expression to amplify and regulate a primary cellular antiviral response that not only serves as a barrier to virus replication, but also functions to educate the innate and adaptive immune systems. Inappropriate activation of antiviral programs can lead to cytotoxicity and cell death. Accordingly, precise regulation of IFN production and response has evolved to prevent inappropriate activation.

Virus induced activation of the IFNβ promoter is known to require the coordinated action of inducible transcription factors at the nucleosome-bounded enhanceosome that recruit chromatin remodeling machinery and allow RNA polymerase activation [2,3]. Several inhibitors and signal attenuators have been identified that can modulate the intensity and duration of IFN signaling and antiviral responses, or re-establish steady state homeostasis following resolution of the infection. One checkpoint in IFNβ expression is provided by diverse histone deacetylase (HDAC) proteins that can mediate either positive or negative regulation [4]. Both HDAC1 and HDAC8 act as repressors of IFNβ gene expression, and depletion of either by RNA interference results in enhanced IFNβ expression due to de-repression [5]. In contrast, HDAC6 acts in a complementary role, to co-activate IFNβ gene expression [5].

In addition to protein-coding genes, recent studies have demonstrated that non-coding RNAs, including endogenous cellular microRNAs (miRNAs), are activated by virus infections and function to modulate mRNA abundance and protein translation [6-13]. MicroRNAs are generated from primary RNA polymerase II transcripts that are processed in the nucleus to create precursor miRNA hairpins. The precursor hairpins are further processed in the cytoplasm to create a mature 17-24 bp miRNA duplex that is incorporated into the RNA-induced silencing complex. Mature miRNAs function to regulate the level of protein production by base-pairing with short seed regions typically within the 3’ UTR of target mRNAs [14-18]. Recognition of mRNA targets by miRNAs can reduce protein expression either by inhibiting target mRNA translation or by promoting target mRNA degradation. Mounting evidence indicates that mRNA destabilization is a predominant means of miRNA-mediated translational repression [19-24]. Although the exact mechanisms and diverse functions of miRNA regulation in innate antiviral immunity are incompletely understood, current information suggests that activation of endogenous miRNA expression enables greater specificity and selectivity in the regulation of antiviral signaling and gene expression.

Several miRNAs have been identified to be important regulators of gene expression during virus infection. A well-characterized miRNA, miR-146a, accumulates during bacteria or virus infections and can negatively regulate cellular signaling molecules including IRAK1, IRAK2, and TRAF6, to disrupt NF-κB activation by TLR and RLR pathways [6,12,25,26]. Another miRNA, miR-132, has also been implicated in both bacterial and viral infections, and can regulate additional antiviral signaling molecules such as the transcriptional co-activator p300 and MAPK3 [11,26]. Cytokine signaling also can regulate miRNA abundance and function, and a group of miRNAs has been demonstrated to increase in abundance in response to IFN stimulation of hepatocytes to limit hepatitis C virus (HCV) replication [10,27]. IFN has also been implicated in regulation of miR-203, which targets the IFN-stimulated gene, ISG56/IFIT1, as well as other pro-inflammatory genes including TNFα and IL-24 [28,29]. These examples demonstrate how miRNAs can contribute in diverse ways to the overall response to virus infection by controlling host gene expression.

Influenza A virus is well known as the causative agent of a contagious respiratory infection and is responsible for seasonal epidemics as well as occasional pandemics, leading to 4,000 to 49,000 deaths per year in the United States alone [30]. Influenza A virus is a member of the *Orthomyxovirus* family of segmented negative strand RNA viruses and is well-documented to inhibit the cellular antiviral responses through a variety of mechanisms including 5’ mRNA cap snatching, RNA sequestration, and inhibition of cellular antiviral signaling pathways [31-33]. More recent studies have indicated that influenza virus infections are able to modulate cellular miRNA and long non-coding RNA expression in macaques, mice, and human cell culture systems, contributing to the regulation of innate and adaptive signaling pathways and their products [26,34-37]. To better understand the extent of miRNA regulation induced by influenza A virus infection, small RNA next-generation sequencing was used to profile the miRNA content of human lung A549 cells before and after infection with strains A/Udorn/72 and A/WSN/33. The mRNA targets for a subset of highly induced miRNAs were identified using a microarray-based screen, and informatics analysis links these targets to broadly regulated cellular networks. To demonstrate the biological consequences of miRNA regulation, a target for the miR-449 family, HDAC1, was analyzed in detail. The highly induced miR-449b was found to target the HDAC1 mRNA, leading to mRNA and protein interference, and subsequent regulation of IFNβ gene expression during stimulation with double stranded RNA and virus infection.

## Materials and Methods

### Cell Culture, Virus Infection, and Transfection

A549 cells (ATCC) were maintained in Ham’s F12 media with Kaign’s modification (F12K, Gibco) supplemented with 10% cosmic calf serum (CCS, HyClone) and 500 units/mL penicillin, and 500 µg/mL streptomycin.

Influenza viruses were grown and titered on Madin Darby Canine Kidney cells. Infections were performed at a multiplicity of 5 plaque forming units (pfu) per cell. A549 cells were washed twice with serum free F12K media prior to inoculation. Influenza virus was diluted to proper concentration in F12K media supplemented with 1% bovine serum albumin. Two hours later, inoculation media was replaced with F12K media supplemented with 2% CCS. RNA was purified 10 hours post inoculation.

Transfections were performed using the following miRNA specific mimics at a final concentration of 50nM: miR-141, miR-147b, miR-190b, miR-199a-5p, miR-374b, miR-449b, miR-512-5p, miR-518b, miR-874, and miR-1263 (Thermo Scientific/Dharmacon). Transfections were performed using Lipofectamine 2000 (Invitrogen) according to manufacturers instructions for RNAi transfection. Flow cytometry was used to determine that 66% of cells were strongly positive for both miRNA and influenza NP, and that 93% of cells that contained the miRNA mimic were also infected.

### Luciferase Reporter Gene Assays

A549 cells were co-transfected with 200ng of -110 IFNβ promoter luciferase reporter gene with 20ng Renilla luciferase control vector to normalize transfection efficiency with or without 50nM miRNA mimic using Lipofectamine 2000 (Invitrogen). Twenty-four hours after transfection, cells were challenged by transfection with 5 µg/mL Poly(I:C) (Invivogen) or infected with Sendai virus (Cantell strain, 5 pfu/cell) for 6 hours. Luciferase activity was measured with the Dual Luciferase Reporter Assay (Promega).

To generate the HDAC1 3’ UTR construct, cDNA from A549 cells was used as a PCR template to amplify 579 bp of the human HDAC1 3’ UTR with the following primers: forward 5’-GCGCGTTTAAACATGGACCTCTCCAGCTCTGG-3’ and reverse 5’-GCGCCTCGAGAGAAATGTACCATTTTATTACAAAGAGGC-3’. The PCR product was cloned into the pMirGLO vector (Promega). Mutagenesis was performed with the QuikChange Lightning Site Directed Mutagenesis kit (Agilent Technologies) according to manufacturer’s protocol with the following primers: forward 5’- CTGGCCTCAAGTGAGCCAAGAAACAGACGGTGCCCTCTGTCTG-3’ and reverse 5’- CAGACAGAGGGCACCGTCTGTTTCTTGGCTCACTTGAGGCCAG-3’. The assay was performed by transfecting A549 cells with 200ng plasmid DNA alone or with 50nM non-targeting control miRNA mimic or miR-449b specific mimic. Twenty-four hours after transfection, luciferase activity was measured with the Dual Luciferase Reporter Assay (Promega).

### RNA Purification and RT-qPCR

Ten hours after infection, RNA was purified and size-fractionated using the Qiagen miRNeasy and minElute Clean-up Kit. The high molecular weight fraction contained RNA >200 nucleotides (nt). The low molecular weight fraction contained RNA <200nt. High molecular weight RNA was used to measure mRNA abundance as in [29] with the following primer sets: IFNβ: forward 5’-CATTACCTGAAGGCCAAGGA-3’ and reverse 5’-CAATTGTCCAGTCCCAGAGG-3’; CCL5: forward 5’-CGCTGTCATCCTCATTGCTA-3’ and reverse 5’-GCACTTGCCACTGGTGTAGA-3’; HDAC1: forward 5’-AAGGAGGAGAAGCCAGAAGC-3’ and reverse: 5’-GTGAGGGACTCAGCAGGAAG-3’; PB1: forward 5’-AATGTGCTAATTGGGCAAGG-3’ and reverse: 5’-CGAATTCTTTTGGTCGCTGT-3’; β-Actin: forward 5’-GGCATCCTCACCCTGAAGTA-3’ and reverse: 5’-AGGTGTGGTGCCAGATTTTC-3’; GAPDH: forward 5’-ACAGTCAGCCGCATCTTCTT-3’ and reverse 5’-ACGACCAAATCCGTTGACTC-3’.

MicroRNA abundance was measured by TaqMan microRNA Assay (Applied Biosystems) as in [29]. Briefly, 10 ng of low molecular weight RNA was reverse transcribed with a miRNA specific primer and MultiScribe reverse transcriptase (Applied Biosystems). Quantitative PCR was performed with a miRNA specific probe according to manufacturer’s instructions (Applied Biosystems). The following TaqMan microRNA Assays were used: hsa-miR-147b, mmu-miR-187, hsa-miR-190b, hsa-miR-449a, hsa-mir-449b, hsa-miR-449c and hsa-miR-874.

### SOLiD Library Construction and Analysis

To construct a library for SOLiD small RNA sequencing, one µg low molecular weight RNA purified from influenza virus infected A549 cells was used in conjunction with the Small RNA Expression Kit (Applied Biosystems) according to manufacturer’s instructions. Emulsion PCR and sequencing was performed at the Northwestern Genetics Core (Center for Genomic Medicine). Analysis was performed using the Applied Biosystems small RNA pipeline software, Bioscope. Briefly, sequence tags were mapped to the human genome with high stringency, no mismatches allowed between the sequence tag and the human genome (hg19 build). These mappable sequence tags were then analyzed to determine RNA classification by BLAST [38]. Sequence tags were then normalized by comparing the frequency of the feature per 1,000 mappable tags [29]. All sequence data are available in the GEO Database under accession number GSE48036.

### Gene Expression Profiling Array and Pathway Analysis

To determine gene expression by microarray analysis, A549 cells were left untransfected or transfected with miRNA specific mimics as described above. Fifteen hours after transfection, cells were either mock infected or infected with A/WSN/33 (5 pfu/cell) for ten hours prior to RNA purification. Gene expression was measured by whole-genome microarray using an Illumina bead array in biological triplicate. Total RNA from three biological replicates was purified 10 hours post infection and hybridized to Illumina Bead Array whole genome expression microarray [39]. Microarray data was analyzed using the Limma software package in R Biocunductor. Samples were normalized by quantile normalization and gene expression differences were determined with a threshold of 1.5 fold change with a p-value of <0.05. Gene expression data are available in the GEO database under accession number GSE47937. Pathway analysis and GO term identification was performed with InnateDB [40,41]. Interactome analysis was generated using Cytoscape with the Agilent Literature Search plug-in [42]. MicroRNA seed match identification was performed using the TargetScan algorithm [43] and the MiRWalk database [44].

### Immunoblot Analysis

To prepare whole cell extracts, A549 cells were washed in ice-cold phosphate buffered saline. Cells were then lysed as in [26] and 10µg of total protein were separated by SDS-Page, transferred to nitrocellulose membrane, and then probed with specific antisera for HDAC1 and GAPDH (Santa Cruz Biotechnologies). Corresponding secondary antibodies conjugated to horseradish peroxidase (Calbiochem) were used. Antibody detection was visualized by chemiluminescence (PerkinElmer) using Vision Works software (UVP). Densitometry analysis was performed by comparing ratios of intensity of HDAC1:GAPDH for each sample using Vision Works software.

## Results

### MicroRNA Regulation by Influenza Virus Infection

**Figure 1:**
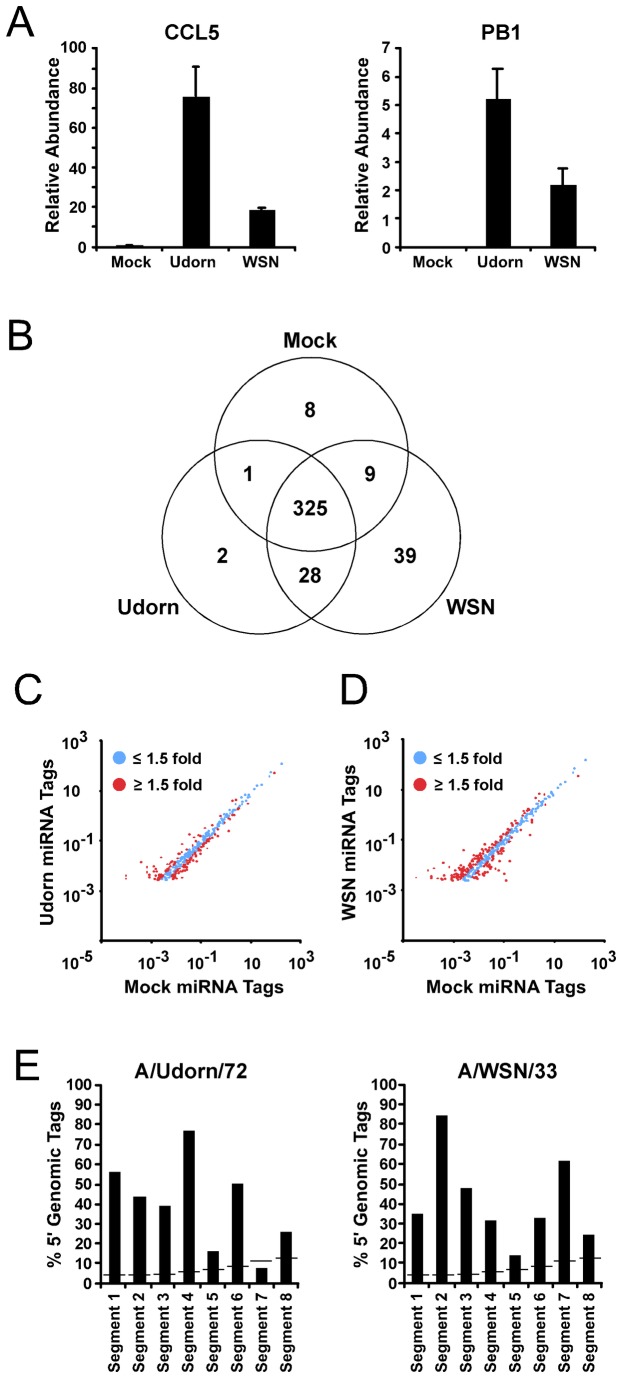
MicroRNA regulation by influenza virus infection. A549 cells were mock infected or infected with either A/Udorn/72 or A/WSN/33 (5 pfu/cell). RNA was purified and size fractionated 10 hours post infection. The low molecular weight RNA was used construct a library for small RNA deep sequencing. (A) High molecular weight RNA was used to measure the expression of the antiviral chemokine CCL5 and the influenza A virus PB1 mRNA by RT-qPCR. (B) Venn diagram indicates the number of common and unique miRNAs identified by a minimum of 100 sequence tags in the indicated sequencing libraries. (C) Comparison of the normalized miRNA sequence tags of the A/Udorn/72 library to the Mock-infected cell library. Blue dots represents miRNAs that exhibit less than 1.5 fold change between libraries. Red dots indicate miRNAs that exhibit greater than 1.5 fold change between libraries. (D) As in (C), comparing the normalized miRNA sequence tags between the A/WSN/33 library and Mock-infected cell library. (E) Histogram indicates the percent of small virally-encoded RNAs that map within 100 nucleotides of the 5’ end of indicated virus genomic RNA segment. The horizontal lines represent the expected percentage of 5’ sequence tags by chance.

To characterize miRNA expression, A549 cells were either mock infected or infected with either of two well-characterized laboratory strains of influenza A virus, A/Udorn/72 and A/WSN/33 (5 pfu/cell). RNA was purified 10 hours post infection and size fractionated to separate high molecular weight RNA (> 200 nucleotides) from low molecular weight RNA (< 200 nucleotides). The high molecular weight RNA was used to ensure that virus infection induced robust cellular antiviral mRNA responses by RT-qPCR, as indicated by induction of the antiviral chemokine CCL5. Additionally, the transcript for the influenza A virus polymerase subunit PB1 was also measured to ensure efficient infection (Figure 1A). The low molecular weight RNA (<200 nucleotides) was used to construct a library for deep sequencing using the SOLiD sequencing platform. The mock infected [29], Udorn, and WSN infected small RNA libraries produced >10^8^ sequence tags, of which >3x10^7^ sequence tags were mapped to the human genome with high stringency (0 mismatches allowed). The mappable sequence tags represent a variety of RNA species including rRNA, snRNA, snoRNA, and miRNA (Table 1). Each library also contained more than 1.5 x 10^7^ sequence tags that represented miRNAs. These sequence tags were further evaluated to determine specific miRNA expression changes during influenza A virus infection compared to steady state. The mock library identified 778 cellular miRNAs, and a similar number of miRNAs was identified in the Udorn (827 miRNAs) and WSN (843 miRNAs) libraries (Table S1). MicroRNAs represented by greater than 100 sequence tags were chosen for further characterization, resulting in 343 miRNAs in the mock library, 356 miRNAs in the Udorn library, and 401 miRNAs in the WSN library. To determine if any gross changes in miRNA expression occurred during infection, miRNAs that were identified by more than 100 sequence tags were compared between samples regardless of their abundance. This analysis identified that 325 miRNAs were identified in all three libraries and that few miRNAs were specific to a given library (Figure 1B). Differential miRNA expression was determined by identifying miRNAs that exhibited greater than 1.5 fold change in normalized sequence tag abundance between the mock infected and influenza A virus infected libraries (Figure 1C,D). Under these criteria, the Udorn library contained 64 miRNAs that increased in abundance and 82 that decreased in abundance, while the WSN library contained 147 miRNAs that increased and 58 that decreased (Table S2).

**Table 1:** Sequence Tag Statistics^**a**^.

	Mock^b^ [29]	A/Udorn/72^b^	A/WSN/33^b^
Total Sequence Tags	97542060^c^	100810000^c^	108804000^c^
Mappable Sequence Tags	32188879	34492704	40087991
microRNA Sequence Tags	18012238	15189854	20848398
miscRNA Sequence Tags	183009	200920	182027
mtRNA Sequence Tags	483546	604482	669441
rRNA Sequence Tags	1297586	1754628	1834363
snoRNA Sequence Tags	1143405	873417	1058720
snRNA Sequence Tags	188817	177575	194572
Other RNA Sequence Tags	11294960	15691828	15300470

a Sequence tag information identifying RNA classes.

b A549 cells were either mock infected or infected with indicated strain of influenza A virus for 10 hours prior to generation of small RNA deep sequencing libraries.

c Number of sequence tags identified in indicated group.

To determine if any small RNAs represented in the sequence tags were generated from the virus genome, all sequence tags that were unable to be mapped to the human genome were aligned to their respective virus genome. This analysis identified 29,523 sequence tags that map to A/Udorn/72 and 15,958 sequence tags that map to A/WSN/33 (Table 2). These small RNAs were largely derived from the virus genome segment 5’ ends (Figure 1E) [45,46], and represent virus-specific sequence tags analogous to the small viral RNAs reported previously for A/PuertoRico/8/34 [46,47], A/Wuhan/359/95, A/HK/54/98, and A/Udorn/72 [45].

**Table 2:** Influenza A Virus Derived Sequence Tags^**a**^.

	A/Udorn/72^b^			A/WSN/33^b^		
	Anti-Genomic^c^	Genomic^c^	Total^c^	Anti-Genomic^c^	Genomic^c^	Total^c^
Segment 1	488	6266	6754	208	1114	1322
Segment 2	341	2212	2553	122	3755	3877
Segment 3	228	1016	1244	138	1925	2063
Segment 4	1700	2087	3787	1037	453	1490
Segment 5	935	1016	1951	1036	412	1448
Segment 6	492	1268	1760	462	661	1123
Segment 7	2091	7283	9374	2398	622	3020
Segment 8	1531	569	2100	1362	253	1615
Total	7806	21717	29523	6763	9195	15958

a Sequence tags unable to be mapped to the human genome were mapped to the indicated influenza virus genome.

b A549 cells were either infected with indicated strain of influenza A virus for 10 hours prior to generation of small RNA deep sequencing libraries.

c Influenza A virus sequence tags that map to indicated segment of genome by RNA sense

### Influenza A Virus Infection Induces Cellular MicroRNAs, Including the miR-449 Family

**Figure 2:**
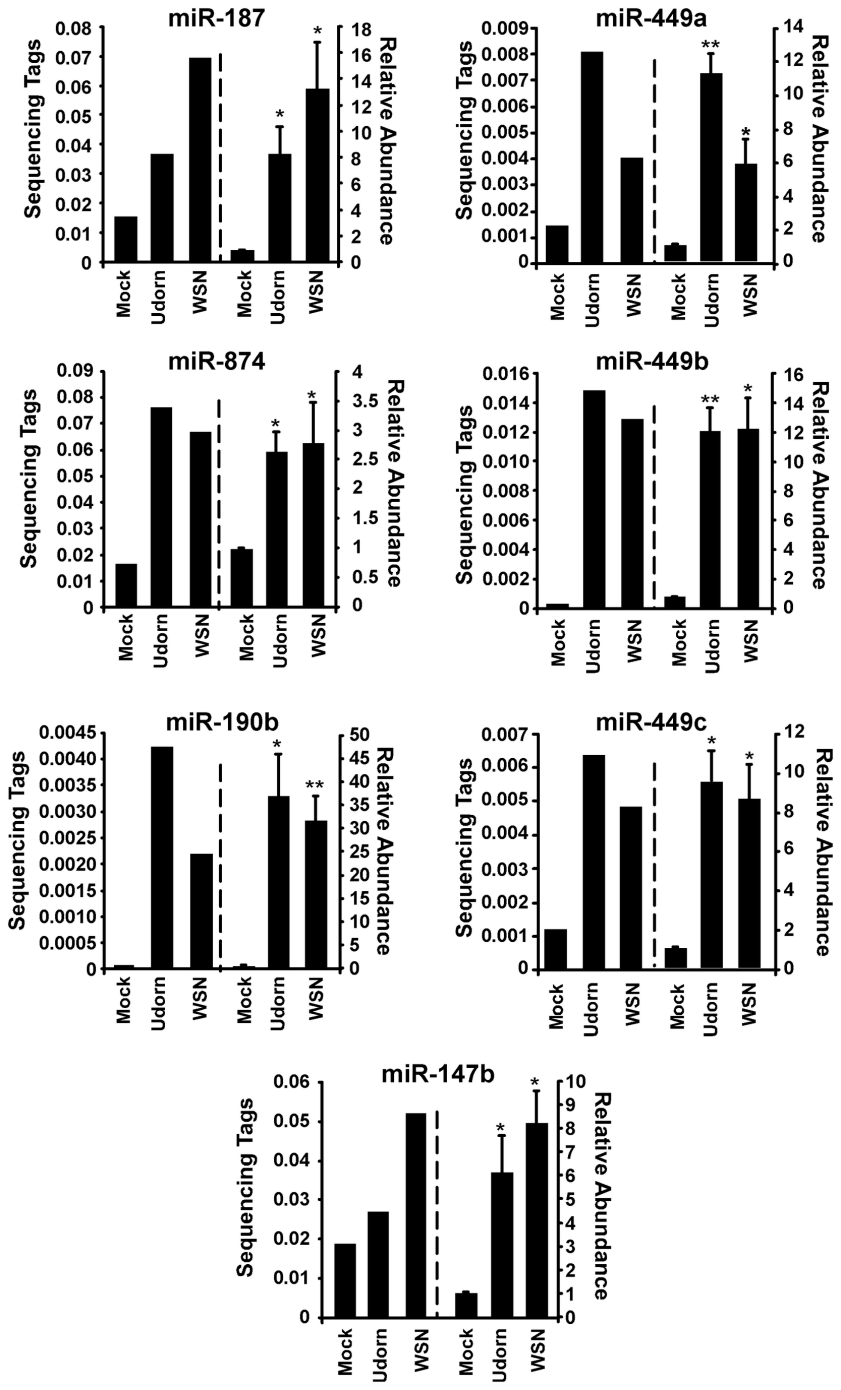
Validation of microRNA activation by influenza A virus infection. A549 cells were mock infected or infected with either A/Udorn/72 or A/WSN/33 (5 pfu/cell). RNA was purified 10 hours post infection and used construct a library for small RNA deep sequencing or TaqMan miRNA RT-qPCR. Data to the left of the dotted vertical line indicate the normalized sequence tags for the indicated miRNA. Data to the right of the dotted vertical line indicate RT-qPCR analysis of the indicated miRNA. Statistical analysis was performed by a two-tailed t-test (p-value<0.05=*, p-value<0.01=**).

The virus-induced expression changes were further verified for a subset of the identified miRNAs using freshly-prepared RNA samples. Total RNA was fractionated to enrich the low molecular weight species, and analyzed by specific RT-qPCR analysis using the TaqMan microRNA Assay system (Applied Biosystems). MicroRNAs miR-187 (previously identified to be induced by influenza A virus [26]), miR-147b, miR-190b, miR-874, and the miR-449 family (miR-449a, miR-449b, and miR-449c) were all validated as highly regulated by influenza A virus infection (Figure 2). Importantly, the results indicate a high correlation between miRNA induction patterns identified by the sequencing analysis and by independent RT-qPCR analysis, with comparable changes in relative expression. These results demonstrate that data from the analysis of sequencing libraries accurately reflect miRNA induction by influenza A virus infection.

### Gene Regulation by Influenza Virus Induced miRNAs

**Figure 3:**
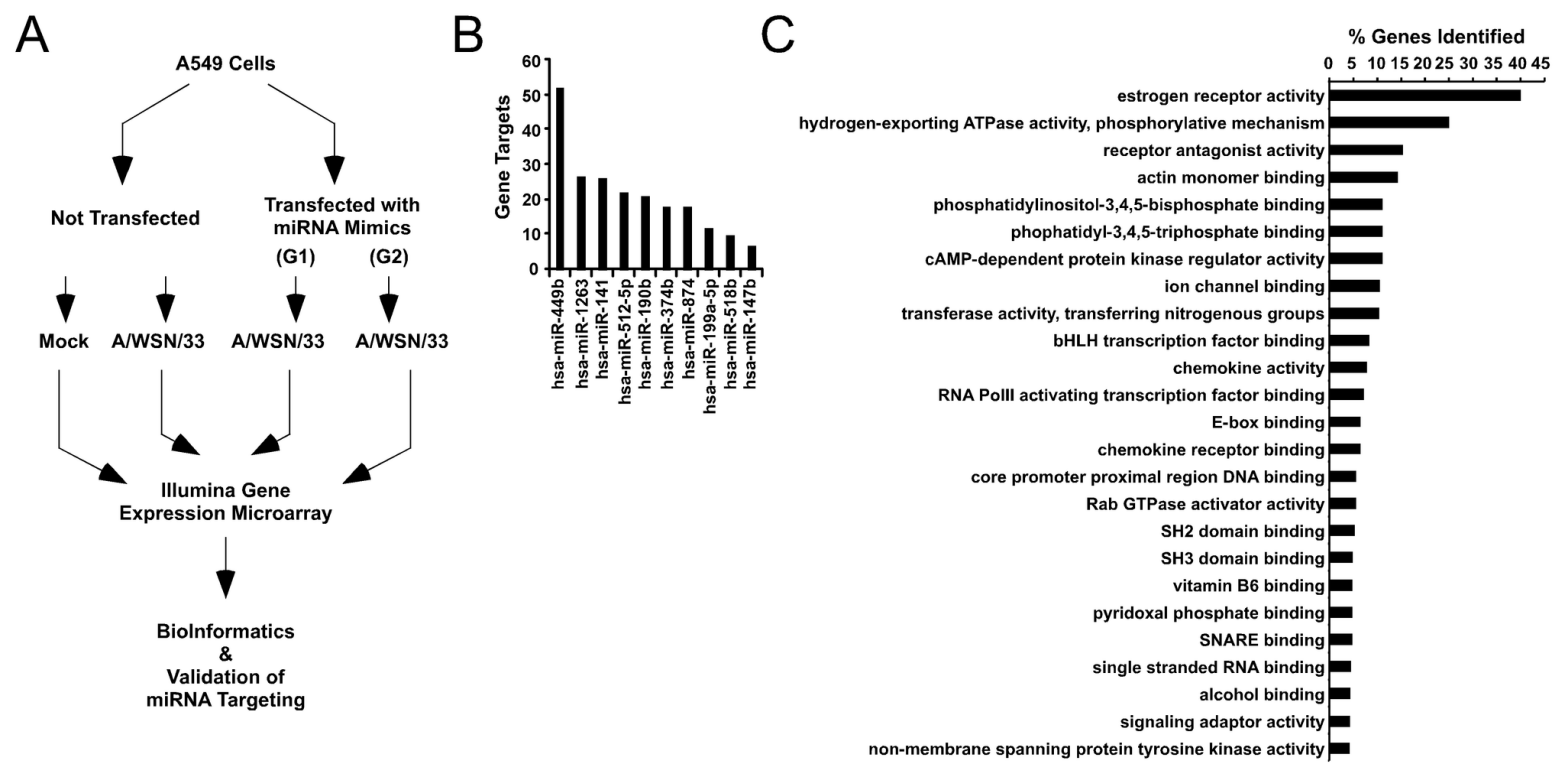
MicroRNA regulation of cellular mRNAs during influenza virus infection. (A) A549 cells were mock infected, infected with A/WSN/33 (5 pfu/cell), or infected after transfection with an equimolar mixture of miRNAs (50nM final concentration) that included either hsa-miR-141, hsa-miR-374b, hsa-miR-449b, hsa-miR-518b, and hsa-miR-1263 (G1), or hsa-miR-147b, hsa-miR-190b, hsa-miR-199a-5p, hsa-miR-512-5p, and hsa-miR-874 (G2). Gene expression profiling was performed by microarray analysis. Genes that exhibited greater than 1.5 fold expression difference and a p-value less than 0.05 were further analyzed. (B) Number of significantly decreased genes that contain a binding site for the indicated miRNA within the 3’ UTR. (C) Molecular function GO terms were identified for down regulated genes based on a literature search with key words “*homo sapiens*,” “infection,” “virus,” and “immunity” to identify known protein interactions with miRNA regulated genes. Histogram depicts the percent of genes identified associated with the indicated GO term.

MicroRNAs function by base-pairing with short seed regions commonly found in the 3’ UTR of target mRNAs, ultimately resulting in mRNA destabilization and degradation [21-23,48]. To identify targets of influenza A virus induced miRNAs, a microarray-based gene expression profiling experiment was performed [26]. A549 cells were transfected with 50nM of a miRNA mimic cocktail to increase their intracellular concentration, and fifteen hours later were infected with A/WSN/33 (5 pfu/cell). RNA was purified 10 h later and gene expression analysis carried out with Illumina bead array whole genome microarrays. Ten highly inducible miRNAs (miR-141, miR-147b, miR-190b, miR-199a-5p, miR-374b, miR-449b, miR-512-5p, miR-518b, miR-874, and miR-1263) were used in this experiment and divided randomly into two groups of 5 for analysis: one group (G1) consisted of miR-141, miR-374b, miR-449b, miR-518b, and miR-1263, and the other group (G2) consisted of miR-147b, miR-190b, miR-199a-5p, miR-512-5p, and miR-874 (Figure 3A). The profiling experiment identified 1976 mRNAs that were differentially regulated by influenza A virus infection, and 170 genes that were affected by miRNA expression during infection by greater than 1.5 fold (Table S3). Of these, 151 were found to decrease in expression level, and 118 of these 151 mRNAs contained predicted target sites for the expressed miRNAs. Among the 118 potentially directly regulated genes, target sites for all expressed miRNAs were identified, with a bias for several specific miRNAs. Target sites for miR-449b, miR-1263, and miR-141 were predicted most frequently in down regulated mRNAs, potentially regulating 52, 27, and 26 mRNAs respectively (Figure 3B).

Informatics and systems biology tools were used to determine the potential relatedness of the miRNA-targeted mRNAs. Gene ontology (GO) terms associated with these genes reflected roles in fundamental cellular pathways related to cell cycle and cytoskeleton regulation (Table S4), and pathway analysis based on the InnateDB database confirms their involvement in fundamental processes related to signal transduction, protein folding, and host-pathogen relationships (Table S5). To further understand how the miRNA targets could be involved in the cellular response to virus infection, an interactome was created based on known connections derived from search terms reflected in the prior literature. The miRNA-regulated genes were subjected to literature search analysis using the search terms “human,” “virus,” “infection,” and “immunity,” a dense network comprised of 658 interconnected genes that could be controlled by the 10 tested miRNAs. The molecular function GO term database was then superimposed on the network to uncover significantly enriched cellular pathways including signal transduction, RNA binding, chemokine actions, transcription regulation, and cytoskeletal dynamics (Figure 3C). These findings support the concept that the G1 and G2 miRNAs function to regulate networks of interconnected genes important for cellular defenses during virus infection.

### HDAC1 Regulation by miR-449b

**Figure 4:**
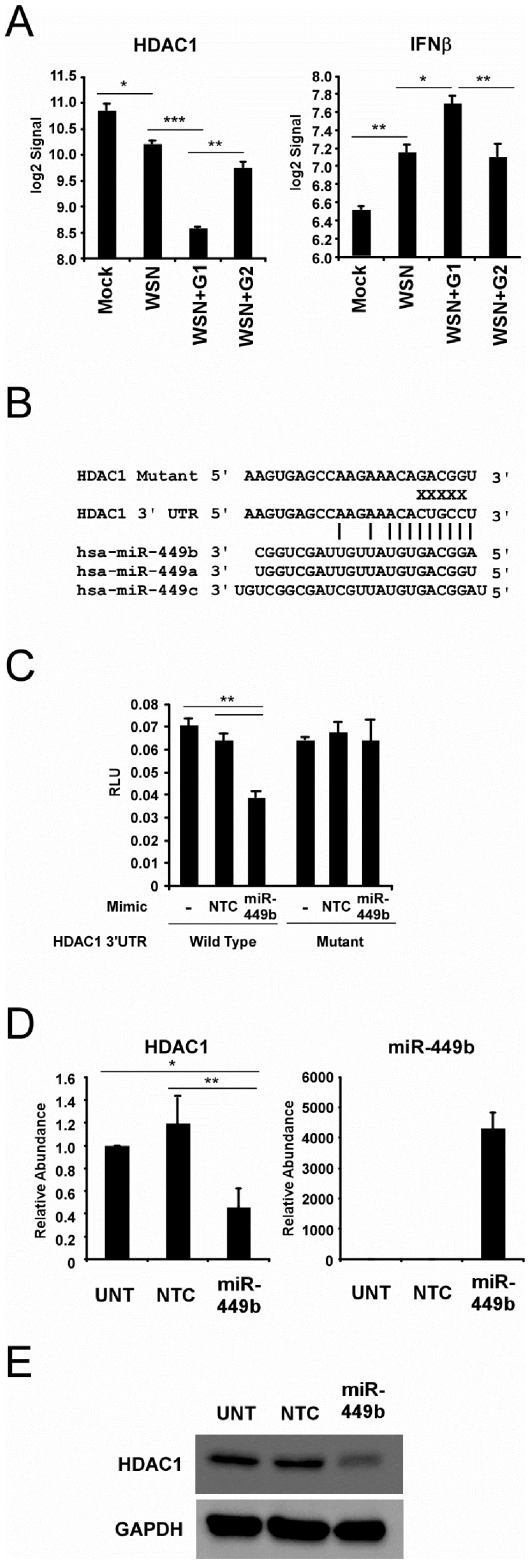
MiR-449b regulates HDAC1 mRNA and protein expression. (A) HDAC1 and IFNβ are regulated by influenza-induced miRNAs. Histograms illustrating the average microarray signal for HDAC1 (left) and IFNβ (right) in the presence or absence of miRNAs described in Figure 3. (B) Depiction of the binding site for miR-449b and other family members in the 3’ UTR of HDAC1 mRNA as determined by TargetScan algorithm [43].. (C) miR-449b directly targets the HDAC1 3’ UTR. A549 cells were transfected with 200ng of luciferase reporter gene containing either the wild type or mutant HDAC1 3’ UTR in the presence or absence of 50nM non-targeting control miRNA mimic (NTC) or miR-449b specific mimic (miR-449b) as indicated. Twenty-four hours after transfection, luciferase activity was measured. (D) miR-449b decreases HDAC1 mRNA level. A549 cells were either untreated (UNT), transfected with 50nM non-targeting control miRNA mimic (NTC) or transfected with 50nM miR-449b specific mimic (miR-449b). Twenty-four hours after transfection, the abundance of HDAC1 mRNA and miR-449b were determined by RT-qPCR. (E) miR-449b decreases HDAC1 protein level. In parallel samples, the abundance of HDAC1 and GAPDH protein was determined by immunoblotting with specific antisera. Statistical analysis was performed by a two-tailed t-test (p-value<0.05=*, p-value<0.01=**, p-value<0.001=***).

To test the veracity of the miRNA target gene identification, the regulation of mRNA targets relevant to antiviral responses was examined in greater detail. Among the differentially-regulated mRNAs identified, two potentially interconnected genes were identified, encoding the histone deacetylase, HDAC1, and the antiviral cytokine, IFNβ (Figure 4A). It was found that the endogenous HDAC1 mRNA level was decreased in response to infection by 2.77 fold in the presence of G1 miRNAs (including miR-449b) during infection, but not G2 miRNAs (Figure 4A). Conversely, cells expressing G1 miRNAs were found to have 68.9% greater IFNβ mRNA compared to infection alone, and 66.5% greater IFNβ mRNA compared to cells expressing G2 miRNAs (Figure 4A). Previous research has demonstrated an essential role for HDAC activity in both positive and negative regulation of the production and response to IFNβ [5,49-53]. Specifically, it has been demonstrated that HDAC1 is required for IFNβ gene repression and siRNA-mediated depletion of HDAC1 results in de-repression of IFNβ gene transcription, producing a higher level of IFNβ in response to virus infection or dsRNA treatment [5]. Examination of the HDAC1 3’-UTR exposes a clearly identifiable seed match for one of the G1 miRNAs, miR-449b, but no miRNAs in G2. MiR-449b shares this conserved seed region with other members of the miR-449 family, miR-449a and miR-449c (Figure 4B), all of which are expressed as a result of influenza A virus infection (Figure 2). These findings suggest a potential regulatory feedback circuit, in which miR-449b could decrease HDAC1 abundance, resulting in an increase of IFNβ promoter activation via de-repression.

To determine if miR-449b is capable of directly regulating HDAC1 expression, a reporter gene was created that fuses the luciferase gene to 579bp of the 3’ UTR of HDAC1. A549 cells were transfected with this reporter alone, or along with 50nM of a non-targeting control miRNA mimic or a miR-449b specific mimic. Luciferase activity was measured twenty-four hours after transfection. The miR-449b mimic significantly inhibits luciferase activity by 39.4% compared to the non-targeting control sample (Figure 4C) verifying the ability of miR-449b to regulate HDAC1 through its 3’ UTR. To determine if miR-449b is using the identified seed region, a variant reporter was created converting the identified 5 nucleotides in the mir-449b seed match to its complement (Figure 4B). Analysis in A549 cells demonstrates that miR-449b is unable to target the mutant reporter, and causes no significant decrease in luciferase activity (Figure 4C). These results indicate that miR-449b directly targets HDAC1 through the identified 3’ UTR seed match, and further validate target identification through gene expression profiling.

To directly test the effect of miR-449b on HDAC1 expression, A549 cells were transfected with either a non-targeting control miRNA mimic or the miR-449b mimic, and total cellular protein and RNA was purified 24 hours later. Analysis by RT-qPCR indicates that miR-449b expression is capable of decreasing steady-state HDAC1 mRNA abundance by 61.4% (Figure 4D). Parallel immunoblot analysis with antisera specific for HDAC1 demonstrates that miR-449b can decrease the level of HDAC1 protein as well (Figure 4E). HDAC1 was readily detected in the cell extract, and the control miRNA had no effect on the abundance of HDAC1. In contrast, expression of miR-449b reduced HDAC1 protein expression level by 46.7% compared to the control miRNA sample (Figure 4E). These results verify the ability of miR-449b to regulate the expression of HDAC1.

### HDAC1 Suppression and IFNβ De-repression by miR-449b

**Figure 5:**
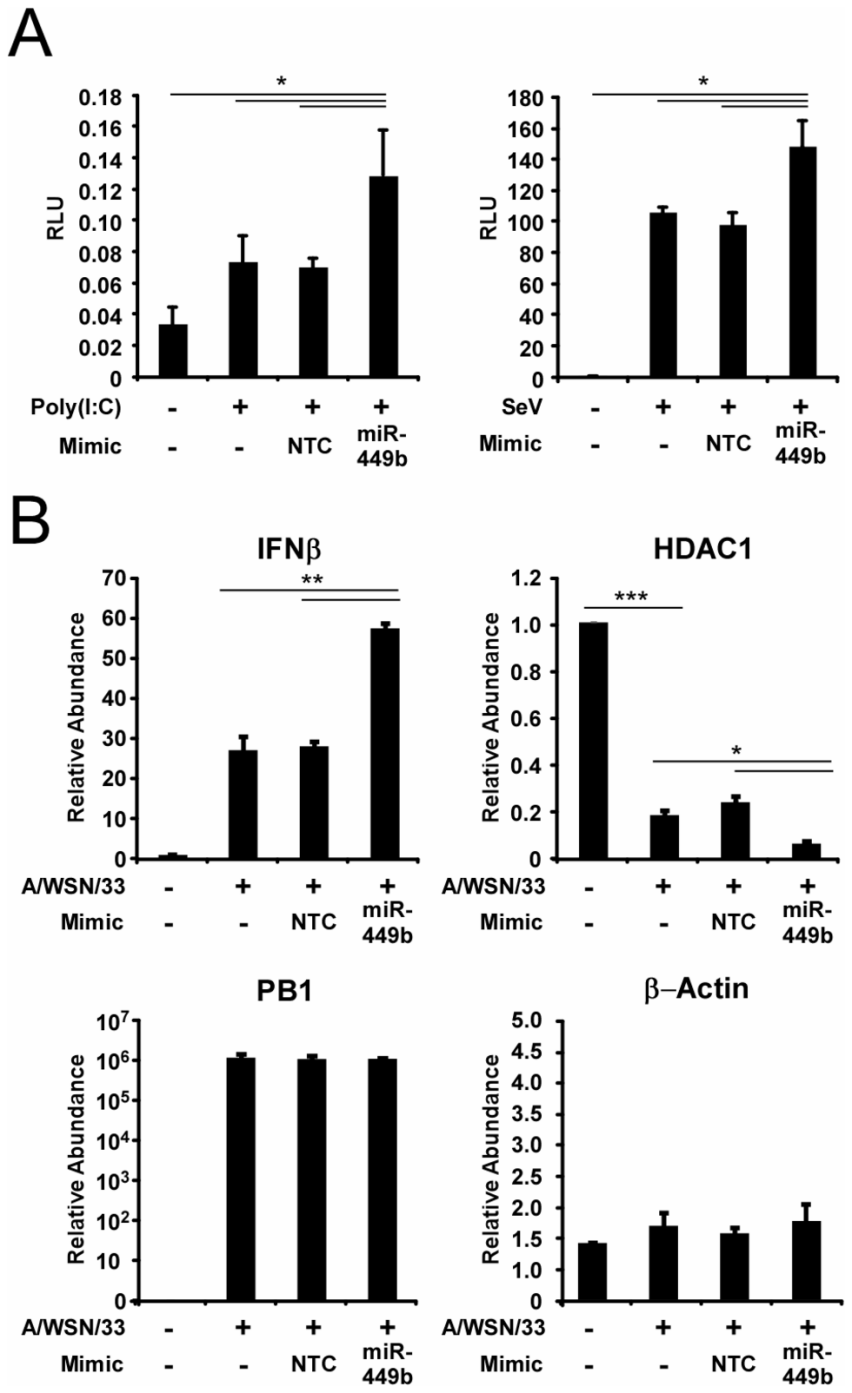
MiR-449b modulates IFNβ expression through regulation of HDAC1. (A) miR-449b derepresses IFNβ promoter activation. IFNβ luciferase reporter gene assay to measure the effect of miR-449b on IFNβ transcriptional activity. A549 cells were transfected with 200ng IFNβ luciferase reporter plasmid and 20ng of *Renilla* luciferase for normalization alone or co-transfected with non-targeting control miRNA mimic (NTC) or miR-449b specific mimic (miR-449b) as indicated. Twenty-four hours after transfection, cells were either left untreated or challenged by Poly(I:C) transfection (5µg/mL) or Sendai virus infection (5 pfu/cell) as indicated for 6 hours prior to measurement of luciferase activity. (B) miR-449b modulates IFNβ mRNA expression during A/WSN/33 infection. A549 cells were either untransfected or transfected with 50nM non-targeting control miRNA mimic (NTC) or miR-449b specific miRNA mimic (miR-449b) as indicated. Fifteen hours after transfection, cells were either mock-infected or infected with A/WSN/33 (5 pfu/cell) for 6 hours prior to RNA purification. The abundance of IFNβ, HDAC1, influenza A virus PB1, and β-actin were measured by RT-qPCR as indicated. Statistical analysis was performed by a two-tailed t-test (p-value<0.05=*, p-value<0.01=**, p-value<0.001=***).

As HDAC1 was previously demonstrated to function as a repressor of IFNβ expression [5], the ability of miR-449b to alter IFNβ expression was tested using an IFNβ promoter-luciferase reporter gene assay. A549 cells were co-transfected with the IFNβ luciferase reporter and control *Renilla* luciferase alone or with a non-targeting control miRNA mimic or miR-449b specific mimic. Twenty-four hours later, cells were stimulated by transfection with the synthetic dsRNA, poly(I:C), or by infection with an IFN inducing strain of Sendai virus, and the luciferase activity was measured six hours later. Expression of miR-449b enhanced IFNβ promoter activation, increasing the poly(I:C)-activated signal by 84.4% and the Sendai virus-activated signal by 54.3% (Figure 5A). Importantly, a control miRNA did not enhance IFNβ promoter activation.

To verify the reporter gene assay, endogenous IFNβ mRNA was measured during influenza virus infection after transfection with miRNA mimics. IFNβ gene expression is induced by 26.8-fold during influenza virus infection and expression of miR-449b increases IFNβ gene expression by 2.15 fold (Figure 5B). As a control, HDAC1 mRNA abundance was measured and verified that influenza virus infection alone decreases HDAC1 mRNA by 81.5% and this decrease is enhanced an additional 35% by miR-449b (Figure 5B). Neither the non-targeting control miRNA mimic nor the miR-449b mimic affected PB1 or β-actin abundance (Figure 5B). These results confirm the relationship between HDAC1 and IFNβ expression, and demonstrate that miR-449b is capable of indirectly enhancing IFNβ expression by directly controlling HDAC1 mRNA and protein levels in the cell.

## Discussion

MicroRNAs are well established as regulators of mRNA stability and translation in diverse cellular processes. This report identifies miRNAs that respond to influenza A virus infection of human A549 cells. The miRNA expression profiling revealed differentially expressed miRNAs that were subsequently used in combination with mRNA microarrays to find potential miRNA-regulated genes. Analysis of a group of 10 virus-induced miRNAs revealed that they are able to decrease the abundance of 151 cellular mRNAs, 118 of which have identifiable 3’ UTR target sites specific for the tested miRNAs. This is likely an underestimation of gene regulation by miRNA expression as small changes in mRNA abundance are difficult to measure using hybridization techniques and any effects on protein translation in the absence of mRNA degradation would be overlooked [54,55]. A computational systems biology approach revealed that these miRNAs could participate in the regulation of a complex network of cellular antiviral responses by affecting pathways such as transcription activation, RNA binding, and chemokine signaling.

Follow-up experiments focused on a biologically relevant miRNA-mRNA pair identified between the miR-449 family and HDAC1. The miR-449 family (miR-449a, miR-449b, and miR-449c) are all encoded within an intronic region of the CDC20b gene on human chromosome 5, and this miRNA cluster was previously reported to be coordinately regulated during airway differentiation and following E2F activation [56,57]. All members of this family share a conserved seed region that can potentially bind to the 3’ UTR of HDAC1 (Figure 4B) and all of these miRNAs have the potential to cooperate to negatively regulate HDAC1, and other targets, during virus infections.

Results demonstrate that miR-449b is capable of regulating the mRNA stability and protein expression level of HDAC1, a protein previously found to be an essential repressive component of the IFNβ enhancer [5]. As a result of this connection between HDAC1 and IFNβ, expression of miR-449b (or its family members) not only interferes with HDAC1 expression, but is also able to enhance IFNβ expression when coupled to antiviral stimulation by poly (I:C) or Sendai virus infection. Expression or inhibition of miR-449b did not have a measurable effect on influenza A virus in assays of replication in cell culture, with no measurable difference in virus titer, plaque size, or growth rates were observed in A549 cells upon miR-449b agonism or antagonism. We speculate that this may in part be due to the many mechanisms that influenza virus has evolved to exploit and inhibit cellular responses, including shutting down host translational machinery and mRNA cap-snatching, as well as diverse interference with innate antiviral signaling and IFN gene activation by NS1. In addition, cellular miRNAs are known to function in fine-tuning biological responses, and the effects of miR-449b may be related to resetting the IFN system, rather than during the peak of highly induced IFNβ during infection.

In addition to the role for miR-449 family members in antiviral responses, a previous report has also implicated miR-449a in targeting HDAC1 in prostate cancer cells. In this context, miR-449a regulation of HDAC1 causes cell cycle arrest and apoptosis [57-59]. Significant morphological changes or cell death were not observed in A549 cells as a result of miR-449b expression during the time course of our experiments, and were possibly obscured by virus-induced cytopathic effects. Nonetheless, these findings highlight the connection between miR-449b and HDAC1, and suggest that additional contextual cues determine its ability to regulate cellular responses in a variety of stress response situations.

The data presented here characterize the miRNA profile of A549 cells before and during influenza A virus infection. Specific changes in miRNA abundance were identified and both bioinformatic and direct analysis implicate miRNAs as important contributors to the cellular responses to virus infection. The miRNA profile and target mRNA analysis further supports growing evidence demonstrating functional roles for miRNAs during influenza A virus infection of human cells.

## Supporting Information

Table S1 **Normalized miRNA Sequence Tags.**
(XLS)

Table S2 **MicroRNAs that exhibited greater than 1.5 fold change during influenza virus infection.**
(XLS)

Table S3 **MicroRNA regulated gene expression changes during influenza virus infection.**
(XLS)

Table S4 **Gene ontology analysis of miRNA regulated genes during influenza virus infection.**
(XLS)

Table S5 **Pathway enrichment of miRNA regulated genes during influenza virus infection.**
(XLS)
